# Bridging the Diagnostic Gap between Histopathologic and Hysteroscopic Chronic Endometritis with Deep Learning Models

**DOI:** 10.3390/medicina60060972

**Published:** 2024-06-12

**Authors:** Kotaro Kitaya, Tadahiro Yasuo, Takeshi Yamaguchi

**Affiliations:** 1 Infertility Center, Iryouhoujin Kouseikai Mihara Hospital, 6-8 Kamikatsura Miyanogo-cho, Nishikyo-ku, Kyoto 615-8227, Japan; 2 Iryouhoujin Kouseikai Katsura-ekimae Mihara Clinic, 103 Katsura OS Plaza Building, 133 Katsura Minamitatsumi-cho, Nishikyo-ku, Kyoto 615-8074, Japan; 3 Department of Obstetrics and Gynecology, Otsu City Hospital, 2-9-9 Motomiya, Otsu 520-0804, Japan; 4 Infertility Center, Daigo Watanabe Clinic, 30-15 Daigo Takahata-cho, Fushimi-ku, Kyoto 601-1375, Japan

**Keywords:** artificial intelligence, chronic endometritis, convolutional neural network, deep learning, infertility

## Abstract

Chronic endometritis (CE) is an inflammatory pathologic condition of the uterine mucosa characterized by unusual infiltration of CD138(+) endometrial stromal plasmacytes (ESPCs). CE is often identified in infertile women with unexplained etiology, tubal factors, endometriosis, repeated implantation failure, and recurrent pregnancy loss. Diagnosis of CE has traditionally relied on endometrial biopsy and histopathologic/immunohistochemistrical detection of ESPCs. Endometrial biopsy, however, is a somewhat painful procedure for the subjects and does not allow us to grasp the whole picture of this mucosal tissue. Meanwhile, fluid hysteroscopy has been recently adopted as a less-invasive diagnostic modality for CE. We launched the ARCHIPELAGO (ARChival Hysteroscopic Image-based Prediction for histopathologic chronic Endometritis in infertile women using deep LeArninG mOdel) study to construct the hysteroscopic CE finding-based prediction tools for histopathologic CE. The development of these deep learning-based novel models and computer-aided detection/diagnosis systems potentially benefits infertile women suffering from this elusive disease.

## 1. Introduction

Chronic endometritis (CE) is an inflammatory pathologic condition of the uterine mucosa characterized by unusual infiltration of endometrial stromal plasmacytes (ESPCs) [1,2,3]. CE is asymptomatic or oligosymptomatic with some subtle symptoms such as vaginal spotting, leukorrhea, and pelvic discomfort. This is in striking contrast to acute endometritis, which manifests with intense clinical symptoms such as systemic fever, pelvic pain, and foul vaginal flow. Due to its silent symptomatology, CE is often overlooked by affected women and even by experienced gynecologists [3].

The principal pathogens of CE are the microorganisms, including common bacteria in the female urogenital organs, Mycoplasma (*M. genitalium* and *M. hominis*), Ureaplasma (*U. urealyticum*), Proteus species, *Corynebacterium*, *Gardnerella vaginalis*, *Klebsiella pneumoniae*, *Pseudomonas aeruginosa*, and yeasts (*Saccharomyces cerevisiae* and *Candida* species) [1,2,3]. *Mycobacterium tuberculosis* is also a microorganism causing granulomatous CE, which is recognized as poorly formed caseating granuloma and lymphoid cell infiltrates containing ESPCs [4]. Antibiotic treatment against these microorganisms is effective in eradicating ESPCs in CE [1,5,6,7].

Meanwhile, the detection rate of *Chlamydia trachomatis* (2–7%) and *Neisseria gonorrhoeae* (0–8%), the representative pathogens causing acute endometritis, is low in the endometrium with CE [1]. Additionally, azithromycin and cefixime, the antibiotics targeting *C. trachomatis* and *N. gonorrhoeae*, have been shown to fail in the preservation of future fertility in women affected by CE [8]. These differences in the microbial profiles between acute endometritis and CE suggest that these two local inflammatory diseases are distinct pathologic entities.

CE is attracting interest because of its association with female infertility, including unexplained etiology (10–57%) [9,10,11], endometriosis (5–53%) [12,13,14,15,16], repeated implantation failure in an in vitro fertilization–embryo transfer program (7–31%) [7,17,18,19,20,21,22,23,24,25,26], unexplained recurrent pregnancy loss (9–13%) [27,28,29,30,31,32], fallopian tubal infertility (45.9% of hydrosalpinx, 14.3% of peritubal adhesion, and 13.0% of tubal occlusion) [33,34,35,36], polycystic ovarian syndrome (32–42%) [37], endometrial polyps (86%) [38,39,40,41,42,43], submucosal uterine fibroids (69%) [44], septate uterus (46%) [44], intrauterine adhesion/Asherman syndrome (28–79%) [44,45,46,47], and cesarean scar disorder (28–66%) [48,49]. The European Society of Human Reproduction and Embryology Working Group recently published Good Practice Recommendations on Recurrent Implantation Failure 2023, a document in which assessment for CE can be considered for infertile women suffering from repeated implantation failure following in vitro fertilization–embryo transfer programs and treatment with antibiotics can be considered if CE is diagnosed in these patients [50]. CE is also thought to be associated with some obstetric complications (preeclampsia and preterm labor) and neonatal diseases in premature infants (periventricular leukomalacia and cerebral palsy) [51,52,53,54,55]. It remains undetermined if the local pathogens deteriorate endometrial receptivity via direct influence on endometrium component cells or if intruding ESPCs (or immunoglobulins and pro-inflammatory molecules produced by these lymphoid cells) pose harm to implanting blastocysts [56].

Currently, universally accepted diagnostic criteria and/or established clinical guidelines do not exist for CE. The diagnosis of CE has traditionally relied on endometrial biopsy and its conventional histopathologic examinations combined with immunohistochemistry (HistoCE) for CD138, an ESPC marker known as transmembrane heparan sulfate proteoglycan syndecan-1 [57]. There are, however, several drawbacks to HistoCE. A somewhat painful procedure (i.e., endometrial biopsy) is required for the subjects. In addition, ESPCs generally accumulate as focal infiltrates (form skip lesions) in CE, so the examinations using the limited volumes of endometrial biopsy samples may miss these landmark cells that are potentially concealed in other mucosal locations. Moreover, there are some concerns that CE can be overdiagnosed with sole immunostaining for CD138 by misidentification of endometrial epithelial cells, which constitutively express CD138 in the plasma membrane [57], as ESPCs. Some studies tested dual immunohistochemistry targeting not only CD138 but also another potential ESPC marker, multiple myeloma oncogene 1, to solve this problem [58,59]. Finally, as researchers have so far defined CE by the threshold or cut-off density of CD138(+) ESPCs based on their original standards or subjective view, there exist many variances and discrepancies in its diagnostic rate among the studies.

Fluid hysteroscopy is emerging as an alternative diagnostic modality for CE. Hysteroscopy is less invasive than HistoCE and enables real-time visualization of whole uterine cavity. Although the potential biases of hysteroscopy are inter- and intra-observer variabilities over the interpretation of the findings, there is more practical consensus on the hysteroscopic diagnosis of CE (HysteroCE) than on HistoCE. In 2019, according to the systematic review and the Delphi poll agreement, the International Working Group for Standardization of Chronic Endometritis Diagnosis proposed the diagnostic criteria of HysteroCE as five representative findings [60]: (1) strawberry aspect [61], (2) focal hyperemia, (3) hemorrhagic spots, (4) micropolyposis [62], and (5) stromal edema. In a randomized controlled observer study, these diagnostic criteria were found to have a positive impact on the ability of reproductive endocrinologists to recognize CE [60].

Deep learning is being actively introduced into the healthcare field. Computer-aided detection/diagnosis systems using deep learning models are often superior to experienced physicians in the identification of lesion areas and the prediction of the probability of the disease [63]. The most advanced algorithms among deep learning models are convolutional neural networks (CNN), a class of artificial neural networks that was recognized at the object recognition competition held in 2012 (ImageNet Large Scale Visual Recognition Competition) and has been dominant in computer vision tasks [64]. A CNN is a hierarchical feed-forward neural network model suitable for large-scale visual recognition with enough neurons to fit any complicated data. CNN models are effective tools for the analysis of medical images, including radiography, computed tomography, magnetic resonance imaging, and endoscopy. Recent progress in the availability of big data and powerful graphics processing units has enabled the training of CNNs. Thus, the integration of the accumulated clinical findings and CNN models has great potential to improve the diagnostic performance of CE.

In 2022, we launched the ARCHIPELAGO (ARChival Hysteroscopic Image-based Prediction for histopathologic chronic Endometritis in infertile women using deep LeArninG mOdel) study. The goal of this study is to link the findings of HistoCE with those of HysteroCE using deep learning-based CNN models and establish computer-aided detection/diagnosis systems for the biopsy-free, less invasive, accurate/precise diagnosis of this inflammatory disease that can deteriorate endometrial receptivity [65]. These approaches could potentially benefit infertile women suffering from CE as well as aid us in establishing diagnostic criteria/clinical guidelines for this yet elusive disease.

## 2. Convolutional Neural Network

### 2.1. Basic Structure of CNN

**Figure 1:**
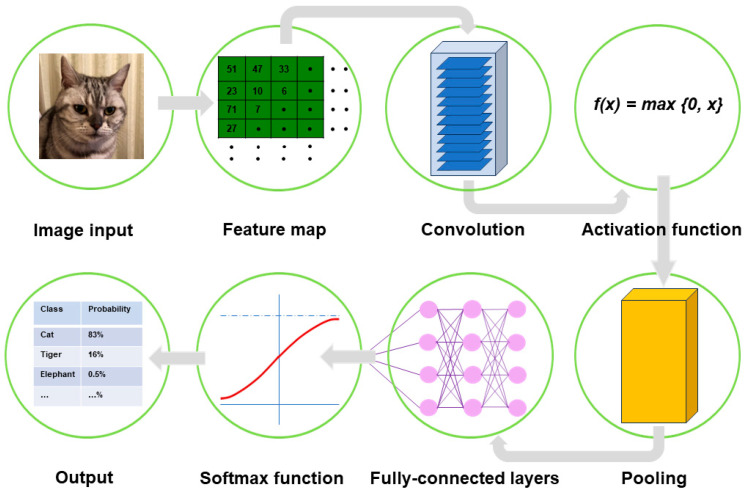
Outline of image processing using convolutional neural network. An image input is converted to pixels, which enables the network to translate the image into a tensor, a grid array of integers ranging from zero to 255. The tensor repeatedly undergoes a convolution process in the hidden layers by multiple kernels, another grid array of integers with an identical size to the window (detailed in Figure 2, Figure 3 and Figure 4), to generate an output feature map that describes the various characteristics extracted from the input tensors. Nonlinear activation functions, represented by the rectified linear unit function, are applied in the convolution process to calculate their individual inputs and their weights. Pooling is performed for downsampling operations to reduce the in-plane dimensionality in the feature map for the introduction of the translation invariance to small shifts, distortions, and a reduction in the number of subsequent learnable parameters. Following the final convolution and/or pooling, the output feature maps are usually transformed into a one-dimensional array of numbers or a vector. These flattened output features are transferred to one or more fully connected layers, where every input is connected to every output by a learnable weight, to set up the final outputs. The softmax function, another activation function, is typically applied to the multiclass classification task for the final outputs of the feature map, resulting in each output value being presented as a probability, ranging from 0 to 1 and summed up to 1. In medical image analysis, this is a benefit both for healthcare providers and patients by presenting the likelihood of normal or abnormal findings or lesions, contrary to healthcare providers only being able to give two answers (i.e., presence or absence).

A typical CNN model comprises a single input layer and an output layer, with an in-between one or multiple hidden layer(s) [66]. The input layer is used as an entrance to capture the images for subsequent processing. Hidden layers perform convolution, which is a specialized type of linear operation that extracts the features from the perceived image inputs and maps them using convolution kernels and an input matrix with activation functions. The created feature map is passed as an input into the following layer. Local or global pooling layers are often added to CNN models to decrease the data dimensions by connecting calculations of neuron clusters from one layer to those of a single neuron in the following layer [67]. Local pooling layers are generally adopted to combine small clusters, whereas global pooling layers engage themselves with all the neurons in the feature map. Two pooling methods are commonly used for CNN models: one is max-pooling, which adopts the maximum value of the local neuron clusters in the feature map, and the other is average pooling, which calculates the mean value in the map [68]. Fully connected layers are put in to connect all the neuron clusters in one layer to those in another layer [69]. Flattened matrices pass through these layers to classify the images for the final output (Figure 1).

### 2.2. Convolution Process

**Figure 2:**
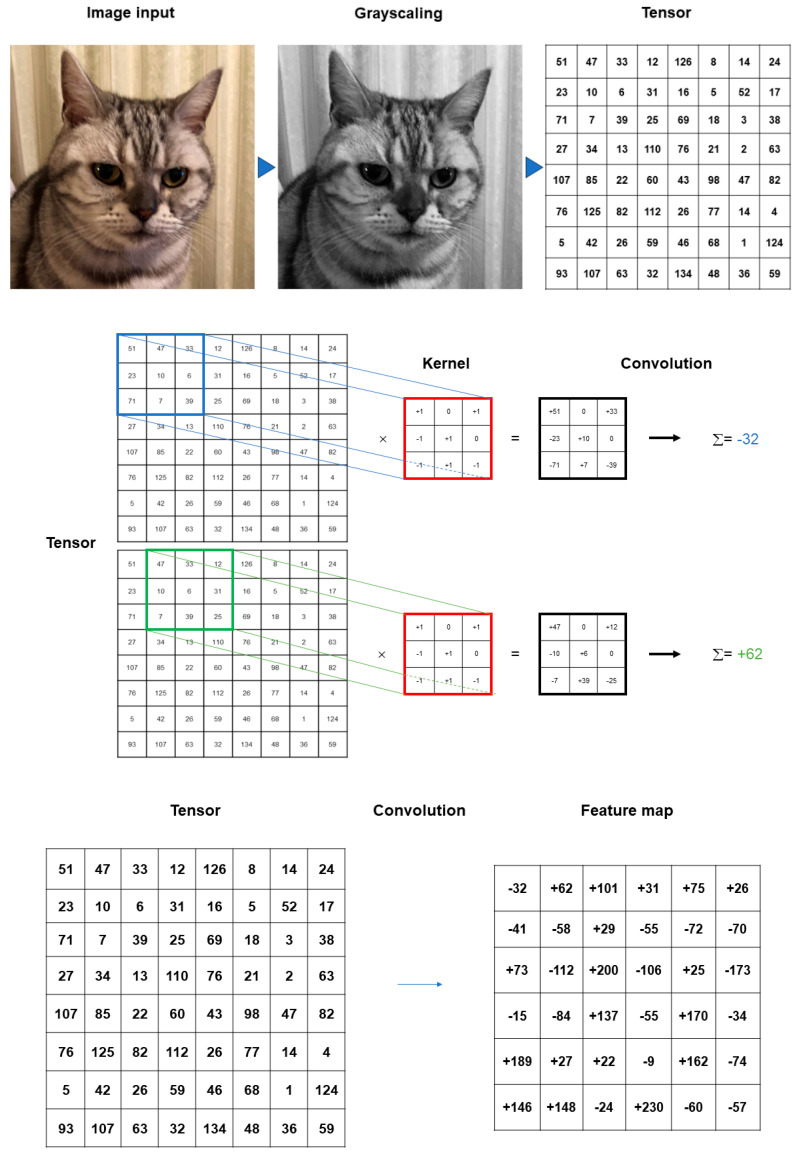
Summary of convolution process. The image inputs are converted to grayscale pixels in advance before the convolution process, by which the values from the primary colors (red, green, and blue) become equivalent. According to the gray tint, the pixels can be deemed as a grid array of integers ranging from 0 to 255 (tensor). An element-wise product is calculated between a window set on the tensor and a grid array of integers, typically with a size of 3 × 3 (kernel). The products are summed up to obtain an output value of an integer at the corresponding position of the tensor. These procedures are repeated by the application of multiple kernels to different windows on the tensor to extract different local features in the tensor, resulting in the creation of a new grid array of integers (feature map).

Convolution is a specialized linear operation that is suitable for feature extraction from image inputs [70]. Prior to the convolution process, the image inputs are typically converted to grayscale pixels, through which the values from the primary colors (red, green, and blue) become equivalent. According to the gray tint, the pixels can be deemed a grid array of integers ranging from zero to 255, termed tensors. In convolution, a window is set on the tensor, and another grid array of integers with an identical size to the window, termed kernel, is applied across the tensor. At each window of a tensor, an element-wise product is calculated between a kernel and a tensor and summed up to acquire an output value of an integer at the corresponding position of the tensor. Repetition of this procedure by application of multiple different kernels to different windows on the tensor extracts different local features in the tensor and creates a new grid array of integers, termed the feature map (Figure 2).

The procedure is repeated with several additional kernels to create an infinite number of output feature maps describing the various characteristics extracted from the input tensors. The size and number of the kernels are the key parameters that define the patterns of the convolution operations. While the kernel size of 3 × 3 is typically used in convolution operations, the kernel number is arbitrary and defines the depth of the output feature maps. In the convolution operations, superimposing the center of each kernel on the outermost element of the input tensor is not allowed, which contributes to the reduction in the height and width of the output feature map in comparison with the input tensor. The procedure for training CNN models is to find the best kernels that can deal with the assigned tasks based on the provided training datasets [70].

Padding is a technique to adapt the center of a kernel to the outermost element and maintain the identical in-plane dimension throughout the convolution operations [71]. Without padding, the size of the feature map can become smaller after each procedure of the convolution operations. Zero padding is most frequently used to fill the void in the outermost element of the input tensor with rows and columns of zeros, which enables the application to more layers.

Stride, which is the distance between two successive kernel positions, is a factor that defines the convolutional operations [72]. The number of the most commonly used stride is one, but strides with two or more are occasionally chosen to obtain downsampling of the feature maps.

Weight sharing is also one of the unique techniques for the convolution operations, where kernels are shared across all the image inputs [73]. The positive effect of the weight sharing is a reduction in model training time and cost by decreasing the number of the weights to be learned as well as making the feature search insensitive to the feature location in the image inputs.

**Figure 3:**
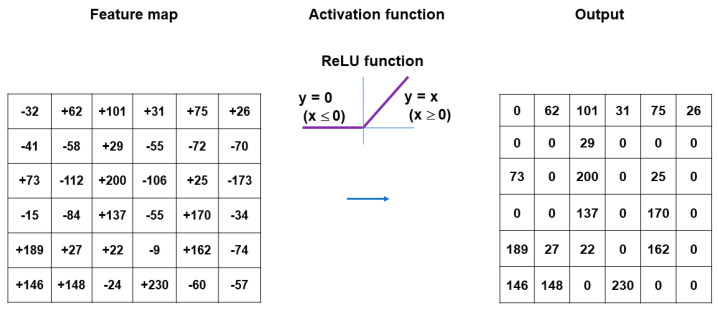
ReLU, defined as f(x) = max (0, x), is a representative nonlinear activation function often adopted in CNN models. x indicates the input to the neuron. ReLU is resistant to the vanishing gradient problem and performs better than the other classical activation functions, such as logistic sigmoid and hyperbolic tangent.

Nonlinear activation functions are used for the outputs of convolution operations [74]. The representative nonlinear activation function adopted in CNN models is the ramp function (rectified linear unit, ReLU), defined as f(x) = max (0, x), where x is the input to a neuron [75]. In the training of CNN models, ReLU was found to perform better than the other classical activation functions, such as logistic sigmoid and hyperbolic tangent (Figure 3). Several efficient methods other than activation functions are being developed to support CNN models for faster convergence and overfitting prevention. These include batch normalization [76], Dropout [77], and DisturbLabel [78].

**Figure 4:**
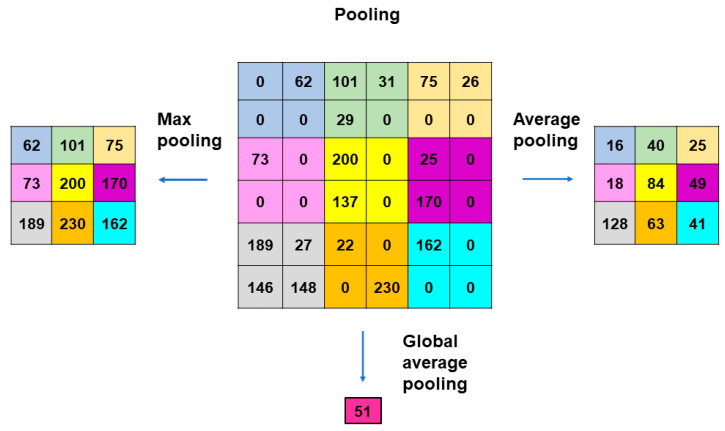
Methods of pooling used in CNN models. Max-pooling is the most popular operation that adopts filters of size 2 × 2 with a stride of 2. Max-pooling contributes to the downsampling of the in-plane dimension in the feature maps by a factor of 2 without altering the depth dimension. Global average pooling is an operation where the average value of all the elements is figured out in each feature map, resulting in the downsampling of the feature map from the size of height × width into a 1 × 1 array. The benefits of global average pooling are a reduction in the counts of the learnable parameters and the implementation of CNN models for accepting inputs with variable sizes. Global average pooling is usually performed only once, immediately before application to the fully connected layers.

Pooling layers give typical downsampling operations to decrease the in-plane dimensionality in the feature maps for the introduction of the translation invariance to small shifts, distortions, and reductions in the number of the subsequent learnable parameters (Figure 4). Max-pooling is the most popular pooling operation in CNN models [70]. A filter of size 2 × 2 with a stride of 2 is most commonly used in max-pooling, which extracts patches out of the input feature maps, outputs the maximum value of the four in each patch and discards the other three values. Thus, max-pooling contributes to the downsampling of the in-plane dimension in the feature maps by a factor of two without altering the depth dimension in the feature maps. Global average pooling is also a pooling operation worth introducing [70]. In global average pooling, the average value of all the elements is figured up in each feature map, resulting in the downsampling of the feature map from the size of height × width into a 1 × 1 array. Meanwhile, the depth of the feature maps is retained in global average pooling. The benefits of global average pooling are a reduction in the counts of the learnable parameters and the implementation of CNN models for accepting inputs with variable sizes. Global average pooling is usually performed only once, immediately before application to the fully connected layers.

### 2.3. Fully Connected Layers and Final Outputs

Following the final convolution and/or pooling operation, the output feature maps are usually transformed into a one-dimensional array of numbers or a vector. These flattened output features are transferred to one or more following fully connected layers, where every input is connected to every output by a learnable weight [69]. The final fully connected layer usually has the same number of output nodes as the number of classes. Each fully connected layer is followed by a nonlinear function. The ReLU function is primarily utilized in these layers.

**Figure 5:**
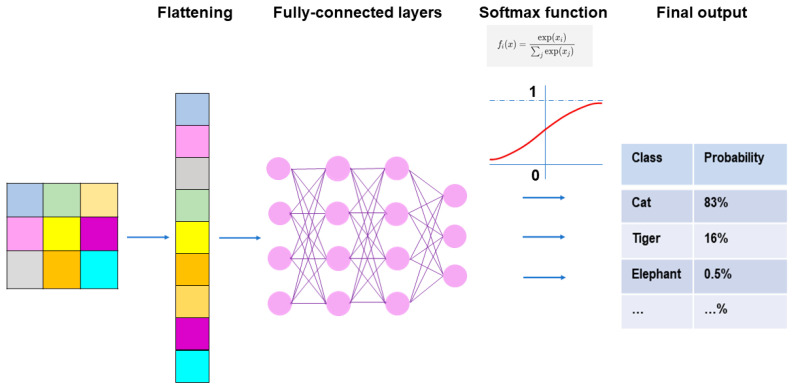
Fully connected layers and final outputs in CNN models. Prior to connecting the pooling layers and fully connected layers, the output of the pooling layers must undergo flattening, which is a process where outputs are transformed from a two-dimensional format into a one-dimensional format that can be understood by the fully connected layers. In the fully connected layers, every neuron in one layer is connected to every neuron in another layer, and the final decisions are made based on the features learned by the CNN. The softmax function is typically applied to the multiclass classification task for the final outputs of the feature maps.

Throughout the fully connected layers, the feature maps are set up for the final outputs. The softmax function, defined as softmax(z_j_) = exp(z_i_)/Σ exp(z_i_), is an activation function that is typically applied to the multiclass classification task for the final outputs of the feature maps (Figure 5) [79]. As the formula of the softmax function contains an exponential conversion of the outputs, the real output values become larger beyond a certain range of numbers. The softmax function works to normalize the real output values passed from the last fully connected layer to target class probabilities, where each output value is presented as a probability, ranging from 0 to 1 and summed up to 1. Thus, the benefit of applying the final outputs to the softmax function is that it can numerically provide the likelihood of abnormal findings or lesions in medical image analysis.

## 3. Application of CNN Model to Hysteroscopic Image Analysis

In parallel with the research and development of novel and diverse CNN models, a myriad of publications is accumulating in the medical field including lesion classification, detection, segmentation, and image reconstruction. Recent significant progress in CNN models for medical image analysis spawned several computer-aided detection systems (such as EndoBRAIN, Olympus, Japan; GI Genius, Medtronic, Ireland; DISCOVERY, Pentax, Japan for colonoscopy; and WISE VISION, NEC, Japan for esophagoscopy) and computer-aided diagnosis systems (such as CAD EYE, Fujifilm, Japan; EndoBRAIN-UC, Olympus, Japan) for endoscopists, which are authorized, commercialized, and applied for clinical practice [80].

Some research groups have introduced CNN models into the image analysis of hysteroscopy for the diagnosis of endometrial pathologic conditions. Török et al. [81] first reported the application of the fully connected CNN model in the identification of the boundaries between the intact myometrium and uterine submucosal fibroid lesion in hysteroscopic surgery. They trained their model with 4688 hysteroscopic images and tested it with 1600 formerly unseen images, achieving an accuracy of 86.19% in hysteroscopic surgery for women with heavy menstrual bleeding and submucosal uterine fibroids.

Burai et al. [82] followed with the development of a CNN model for the automated assessment of the uterine wall in infertile women undergoing in vitro fertilization–embryo transfer treatment. They used an aggregation for the overlapping legions of the sub-images, which have been generated from the splitting of the original images. In addition, the segmentation results of the multiple CNNs were combined via a weighted combination model and optimized for adjusting the weights. The accuracy of the model reached 91.56% regarding the recognition of the uterus wall.

Sun et al. [83] substantiated the availability of their hierarchy framework HIENet (Bidirectional Hierarchy Framework for Automated International Classification of Diseases Coding) model, a computer-aided diagnosis-oriented CNN containing three main modules, including progressive mechanism, bidirectional hierarchy passage encoder, and personalized PageRank, along with attention mechanisms [83]. HIENet was capable of classifying the microscopic images of the conventionally stained endometrial preparations into nonpathological, polyp, hyperplasia, and adenocarcinoma. Following the testing, training, and further cross-validation, their model achieved an accuracy of 0.845 and an area under the curve value of 0.9829 with 77.97% sensitivity and 100% specificity, which outperformed three expert pathologists and the other five existing deep learning-based classification algorithms.

Meanwhile, Zhang et al. [84] established an Oxford Visual Geometry Group network (VGGNet)-16/ImageNet-based CNN model that can perform the automatic classification of the endometrium-associated lesions presented by inputs of hysteroscopic images into polyps, submucosal fibroids, hyperplasia without atypia, atypical hyperplasia, and cancer. Developed by the Oxford Visual Geometry Group [85], VGGNet-16 comprises the convolution layers (3 × 3 size of kernels) and the max-pooling layers (2 × 2 size of kernels) with a total of 138 million parameters. Following testing, training, and validation, their model finally achieved an accuracy of 0.808 and outperformed multiple expert gynecologists with 68.0% sensitivity and 95.5% specificity for atypical hyperplasia, and 78.0% sensitivity and 96.5% specificity for endometrial cancer, respectively. In the classification task of endometrium-associated lesions into two categories (benign or premalignant/malignant), the model performance eventually reached 90.8% accuracy, 83.0% sensitivity, and 96.0% specificity, respectively.

Takahashi et al. [86] disclosed an automated hysteroscopic image analysis CNN system that can identify endometrial cancer lesions from normal endometrial tissues. Xception (originally known as Extreme Inception) is one of the versions in the Inception family [87]. Xception is constructed based on the inception module with modifications and combinations with convolutional layers, depth-wise separable convolutions, and residual connections, resulting in superior classification performance to VGGNet. MobileNetV2 is a bottleneck-depth separable CNN architecture with two three-layer basic blocks with residuals [88]. The layers consist of 1 × 1 convolution with ReLU and stride 1, depth-wise convolution with stride 2, and 1 × 1 convolution with no non-linearity. EfficientNet-B0 is a CNN architecture utilized as an encoder in semantic segmentation tasks [89]. EfficientNet-B0 has been adopted as a backbone network to extract the features out of the input image by downsampling. Using the cascades of Xception, MobileNetV2, and EfficientNetB0, their system obtained a higher accuracy of 90.29% with 91.66% sensitivity and 89.36% specificity, respectively, of which scores were statistically higher compared with the conventional human diagnostic systems (78.91–80.93%).

Air embolism is one of the potentially serious complications during hysteroscopy. U-net is an image segmentation technique widely adopted in major image modalities, ranging from radiography, computed tomography, magnetic resonance imaging, and microscopy [90]. To develop the automated air bubble removal devices in hysteroscopy, Wang et al. [91] generated a unique U-Net-based CNN model named edge-aware network that is equipped with two branches and combined with a marker-controlled watershed system. The main segmentation branch is aimed at merging multiscale features and predicting segmentation results, whereas the role of the contour branch is the prediction of the bubble boundary and the fusion of the output with the main segmentation branch. Moreover, the auxiliary edge supervision mechanism is attached to enhance the segmentation performance and overcome the deficiencies in information on the blurry bubble boundaries. The edge-aware network model performed better than some typical segmentation methods, with an accuracy of 0.859 ± 0.017, a sensitivity of 0.868 ± 0.019, a precision of 0.955 ± 0.005, a Dice score of 0.862 ± 0.005, and a mean intersection over union 0.758 ± 0.007. Thus, the edge-aware network model has potential that is feasible for the development of automatic bubble removal devices in hysteroscopic surgery.

Object detection algorithms are emerging as new tools for medical image analysis in power systems because of their excellent performance in generalization capability and feature extraction of objects from complex backgrounds. Object detection algorithms are typically subdivided into two categories. One is the two-stage algorithm, and the other is the one-stage algorithm. Two-stage algorithms usually adopt region proposal networks to decrease the interference in the complex backgrounds of the insulator detection and defect detection. While the precision of insulator detection is improving gradually, the low efficiency and slow speed have been challenging in medical image analysis [92]. The You Only Look Once (YOLO) series are the representative one-stage object detection algorithms, which eliminate the region proposal network, generate and set the position coordinates, and calculate the category probability of the object in a single detection operation [93]. The architecture of the YOLO series is characterized by an anchor-free design and decoupled head structure, which are superior in real-time object detection in images. The evolution of the YOLO series enabled the one-stage object detection operation to finish the tasks quickly and accurately. YOLOX is the developed version of the YOLO series. Although YOLOX inherits the low accuracy problems in the presence of insulator defect detection as well as the vulnerability of the detection results to interference by complex backgrounds, YOLOX has faster speed and higher accuracy in object detection compared with the ancestral versions [94]. Zhao et al. [95] adopted a modified YOLOX model for accurate identification of endometrial polyps in hysteroscopic video images. They combined the model with the group normalization method, of which characterization lies in the calculation of the mean and standard deviation for normalization using the set of pixels based on four-dimensional vectors (batch axis, channel axis, and spatial axes of height and width). The strength of this model is its availability for small batch sizes without compromising the performance of the model. Moreover, to overcome the problems of the unstable object detection boxes in the detection task of the endometrial polypoid lesions, they added a perceptual hashing-based video adjacent-frame association algorithm to the post-processing stage [96]. Perceptual hashing is a one-way mapping method that enables the mapping of multimedia data with perceptual content to an identical digital digest and enhances the robustness and security of perception. In the video adjacent-frame association algorithm, the similarity between adjacent frames is evaluated and applied for the stabilization of the detection boxes in similar frames. These operations were found to aid in the stabilization of the detection boxes and the improvement in the accuracy of the object detection results. After training with 11,839 images from 323 cases, their modified model achieved higher sensitivity (100% and 92.0%) in the detection of hysteroscopic endometrial polyps for the two test datasets of 431 cases, compared with 95.83% and 77.33%, respectively, by the original YOLOX model.

In addition to hysteroscopic image analysis, using a high-performance anchor-free version of YOLOX, Jiang et al. [59] developed a deep learning-based trained CNN model for the automated detection of ESPCs in the photographed images of endometrial specimens with dual immunostaining for CD138 and multiple myeloma oncogene-1 (MUM1), another marker for plasmacytes. Following the identification of the valid areas according to the sliding window-based cropping of the whole slide images, a total of 2000 regions of interest were chosen as the testing dataset. Residual neural network 18 (ResNet18) is a deep residual neural network that replaces the convolution and pooling layers of CNN with fully connected layers [97]. ResNet18 consists of one input layer, one output layer, and in-between three sets of one dense block and two identity blocks with three hidden dense layers in each block. While the input is connected to the output via another dense layer in a dense block, it is also directly connected to the identity blocks. Meanwhile, extreme gradient boosting (XGBoost) is an optimized distributed gradient-boosting library based on binary decision tree algorithms that exert machine learning under the gradient boosting decision tree framework [98]. XGBoost operates on the second-order Taylor approximation of the cost function and takes advantage of the results of both the first and second derivatives. While XGBoost was adopted to deal with the extracted shape, color, and texture features of ESPCs, ResNet18 was added as a post-processing module to lessen the false positive rates and enhance the precision value in this study. This cascade model finally achieved sensitivity, specificity, and accuracy rates of 100%, 83.3%, and 91.4%, respectively, for HistoCE. The model was found to distinguish CD138(+)/MUM1(+) ESPCs from false-positive cells that are immunostained with either CD138 alone (14%) or MUM1 alone (24%), suggesting that it has the potential to prevent overdiagnosis of HistoCE. When the threshold of five or more CD138(+)/MUM1(+) ESPCs per section was set for the diagnosis of CE, all the scores of sensitivity, specificity, and accuracy reached 100%, indicating the superiority of the combination of the dual immunohistochemistry for CD138/MUM-1 and deep learning model to that of the single immunohistochemistry for CD138 and visual observation/manual counting by pathologists in HistoCE. The development of these kinds of models holds promise for the establishment of the definition of the optimal threshold/cut-off ESPC density for HistoCE, which is another important unsolved problem in CE.

## 4. Application of CNN Model to HysteroCE

### 4.1. Application of CNN Model to Diagnosis of Endometrial Micropolyposis

**Figure 6:**
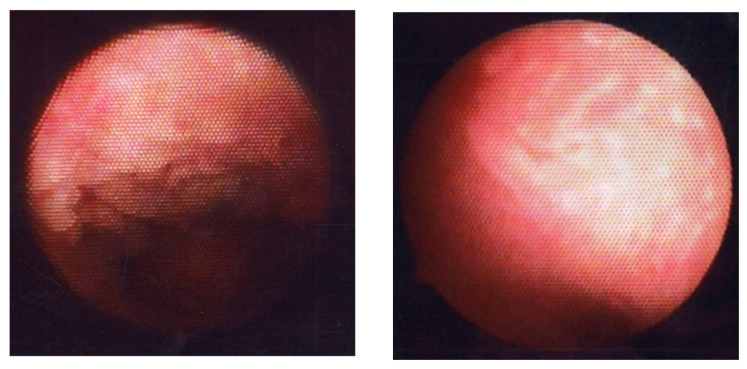
Fluid hysteroscopic images of endometrium with CE. Micropolyposis (**left**) and strawberry aspect (**right**).

Of the five representative HysteroCE findings described above [60], endometrial micropolyposis (a cluster of typically less than 1 mm sized protrusions on the whole or focal mucosal surface, Figure 6, left panel) is the most studied and well-characterized one so far, following the first report by Cicinelli et al. [62]. They found micropolyposis in 11.7% of women undergoing hysteroscopy due to any gynecologic indications. Micropolyposis unexceptionally coexisted with other HysteroCE findings. In 93.7% of women with micropolyposis, HistoCE (based on ESPC infiltration) was identified with conventional tissue staining, in striking contrast with only 10.8% of women without micropolyposis having HistoCE. Considering all cases of HistoCE, micropolyposis was detected in 53.6% of the cases. Overall, they concluded that the diagnostic accuracy for HistoCE in the presence of micropolyposis rises to 90% (sensitivity 54%, specificity 99%, positive predictive value 94%, and negative predictive value 89%). Zolghadri et al. [28] also reported micropolyposis (in combination with or without hyperemia) as a hysteroCE finding with higher sensitivity (98.4%) and negative predictive values (97.82%), but with lower specificity (56.23%) and positive predictive values (63.5%) to predict the presence of HistoCE. The bias and limitations of these studies were that HistoCE was diagnosed with endometrial conventionally diagnosed specimens only.

Using both hysteroscopy and immunohistochemistry for CD138, we retrospectively investigated the association between micropolyposis and HistoCE (defined as ≥0.25 CD138(+) ESPCs per HPF) in infertile women with a history of repeated implantation failure [99]. The sensitivity, specificity, positive predictive value, negative predictive value, and diagnostic accuracy of the hysteroscopic detection of endometrial micropolyposis to predict the presence of HistoCE were 65%, 66%, 60%, 70%, and 65%, respectively. Intriguingly, in striking contrast to the high rate of concomitance of HistoCE and micropolyposis, the prevalence of HistoCE was only 4% in a cohort of infertile women with classical endometrial polyps in this study. The biases and limitations of this study were its small sample size and retrospective design.

Song et al. [100] enrolled a larger cohort of 1189 premenopausal women to discover HysteroCE findings relevant to HistoCE, which was defined as ≥0.1 CD138(+) ESPCs per HPF. With this threshold, the sensitivity, specificity, positive predictive value, negative predictive value, and diagnostic accuracy were 59.3%, 69.7%, 42.1%, 82.8%, and 66.9%, respectively, when any one of micropolyposis, hyperemia, and edema was detected. If the presence of two or three of these hysteroscopic CE features was adopted, the specificity (99%) and positive predictive value (64%) increased significantly. Still, the sensitivity (5%) and negative predictive value (73.3%) fell, which increased diagnostic accuracy (73.5%). Again, the potential bias of this study was its retrospective design.

Bouet et al. [101] prospectively investigated micropolyposis (in combination with hyperemia) as a potential factor to predict the presence of HistoCE in 94 infertile women with a history of repeated implantation failure in an in vitro fertilization program and/or unexplained recurrent pregnancy loss. When the threshold of ESPC density for HistoCE was set as ≥0.5 CD138(+) ESPCs per HPF, the sensitivity, specificity, positive predictive value, and negative predictive value scored 40%, 80%, 35%, and 83%, respectively, resulting in an accuracy of 71%. As shown in the aforementioned studies, micropolyposis often coexists with other HysteroCE findings, such as stromal edema and hyperemia in an individual. A recent meta-analysis demonstrated that micropolyposis, stromal edema, and hyperemia are predominant features of five HysteroCE findings in infertile women [102,103]. The diagnostic accuracy of micropolyposis (alone or combined with stromal edema and/or hyperemia) on hysteroscopy for the prediction of the presence of HistoCE is estimated between 60% and 70% [104].

There are currently no deep learning models that focus on aiding the identification of HysteroCE. Using archival hysteroscopic images obtained from women with HistoCE (defined as ≥0.25 CD138(+) ESPCs per HPF [99]) on days 6–12 in the menstrual cycle, we aimed to construct a VGGnet-16-based CNN model for the automatic detection of micropolyposis [105]. The images were manually processed to retain the lesions of interest and eliminate the confusing sites to enhance the performance of the CNN model. The data augmentation was artificially undertaken to increase the number of training sets and the robustness of the model against predictable variations, such as imaging artifacts and noise. The images, excluding poor quality, massive uterine bleeding, mixed intrauterine lesions, intrauterine device, and malignancy/hyperplasia, were initially reviewed by three experienced gynecologists/reproductive endocrinologists/endoscopists who were recruited as a reference in the comparison with the CNN model for assessment of micropolyposis. The model was trained on ImageNet with the addition of a batch normalization layer following each convolutional layer for the improvement in the training speed. A root mean square propagation optimizer, an adaptive learning rate method that originates from the extension of the stochastic gradient descent algorithm and momentum method with the momentum 0.9, initial learning rate 1 × 10^−1^, and end learning rate 1 × 10^−4^, was also adopted for the minimization of the binary cross entropy loss function and scoring the best accuracy value [61,62,63]. The parameters that resulted in the best performance were selected for the final pieces for the prevention of overfitting. The analysis of the archival hysteroscopic images of 208 infertile patients with HistoCE who met the inclusion criteria demonstrated that micropolyposis was identified in a total of 37.5% of the subjects, whereas HistoCE was identified in 28.8%. The sensitivity/specificity of the conventional diagnosis with micropolyposis for a test dataset by three experienced gynecologists were 94.9%/93.1%, 92.3%/94.6%, and 91.0%/90.1%, respectively, whereas those values by our CNN model were 93.6%/92.3%, respectively. These findings resulted in the accuracy, precision, and an F1-score of the conventional diagnosis by these gynecologists (93.8%/89.2%/0.919, 93.8%/91.1%/0.917, and 90.9%/85.5%/0.882) and our CNN model (92.8%/88.0%/0.907), respectively. Finally, the area under the receiver operating characteristic curves of the conventional diagnosis by three gynecologists was 0.940, 0.935, and 0.906, whereas that by our CNN model was 0.930, respectively. Thus, the diagnostic performance of micropolyposis by the current CNN model-aided system reached a similar level (*p* > 0.05, DeLong test) to that of experienced gynecologists.

### 4.2. Application of CNN Model to Diagnosis of Strawberry Aspect

Strawberry aspect (scattered large hyperemic areas flushed with white central points, Figure 6, right panel) is a HysteroCE finding that was described earlier by Cravello et al. [61]. Bouet et al. [101] reported that strawberry aspect is identified in 65% of infertile women undergoing repeated implantation failure in an in vitro fertilization program and/or unexplained recurrent pregnancy loss with HistoCE. There was a positive correlation (16–54% for sensitivity and 60–94% for specificity) between the presence of strawberry aspect and HistoCE when the finding was combined with other HysteroCE findings including micropolyposis. They also discussed that the lesions with strawberry aspect are unique to CE but are so mild that they may be potentially overlooked [101]. Wang et al. [106], detected strawberry aspect in 43.4% of infertile women with a history of repeated implantation failure in an in vitro fertilization program, which was confirmed with HistoCE (using conventional hematoxylin and eosin tissue staining, but not by immunohistochemistry for CD138). They also found that the expression level of interleukin-17 [107], a proinflammatory cytokine, in the endometrium, was higher in women with HistoCE than in those without HistoCE, whereas local expression of interleukin-10 [108], an anti-inflammatory cytokine, and transforming growth factor-β [109], a master regulator of inflammation, was lower in CE. Tsonis et al. [104] described that the diagnostic accuracy of the presence of micropolyposis, stromal edema, hyperemia, and strawberry aspect for HistoCE is 99.75%, 82.35%, 94.95%, and 94.77%, respectively, and agreed that strawberry aspect is a unique HysteroCE finding that potentially predicts the presence of HistoCE.

Clinical information on strawberry aspect is, however, scarce compared with micropolyposis [110]. We are investigating the characteristics of HistoCE with strawberry aspect. In contrast to micropolyposis being often detected without other HysteroCE findings (approximately 60% of HistoCE cases), strawberry aspect was usually concomitant with any of micropolyposis, stromal edema, hyperemia, and/or hemorrhagic spots (more than 95% of HistoCE cases). In addition, more focal ESPC infiltration was seen in HistoCE with strawberry aspect (unpublished observation). These findings suggest that CE may be subtyped by the degree of the HysteroCE and ESPC infiltrates, although further studies are indispensable for confirmation.

## 5. Conclusions

The ARCHIPELAGO Study is still in its infancy, as the CNN models for identification of strawberry aspect are under construction and those for other HysteroCE findings are yet untouched. These approaches are, however, promising to bridge the diagnostic gap between HistoCE and HysteroCE as shown in detection of micropolyposis, although the effectiveness and generalizability of the model need to be rigorously validated across diverse patient populations and healthcare settings. The further development of these models will enable less-invasive diagnosis of CE and will potentially benefit infertile women suffering from this elusive disease.

## Data Availability

Not applicable.
